# Sociodemographic differences in the use of dietary supplements in a representative sample of adults in Poland—a secondary analysis

**DOI:** 10.3389/fnut.2025.1724264

**Published:** 2025-12-16

**Authors:** Radosław Sierpiński, Mateusz Jankowski, Filip Raciborski, Agnieszka Kamińska

**Affiliations:** 1 Faculty of Medicine, Collegium Medicum, Cardinal Stefan Wyszynski University, Warsaw, Poland; 2 Department of Population Health, School of Public Health, Centre of Postgraduate Medical Education, Warsaw, Poland; 3 Department of Prevention of Environmental Hazards, Allergology and Immunology, Faculty of Health Sciences, Medical University of Warsaw, Warsaw, Poland

**Keywords:** diet, dietary supplementation, nutritional behaviors, sociodemographic factors, public awareness, Poland

## Abstract

**Introduction:**

Dietary supplements are concentrated sources of vitamins, minerals, or other substances with nutritional or physiological effects. This study aimed to assess sociodemographic differences in the use of dietary supplements in a representative sample of adults in Poland.

**Methods:**

This is a secondary analysis of a dataset generated during the representative cross-sectional survey (December 2024) carried out among 5,006 adults aged 18–64 years in Poland. Attitudes towards the use of dietary supplements were assessed.

**Results:**

Among all respondents (*n* = 5,006), 39.1% reported regular use of dietary supplements in the 3 months preceding the present study, and another 31.5% reported occasional use. Among those who used supplements in the 3 months preceding the study, 11.4% had all of their supplements prescribed by a doctor, and another 22.7% had some of them. The highest prevalence of dietary supplement use was among those following a low-carbohydrate diet (58.4% regular and 27.9% occasional use), and the lowest among those who reported not paying much attention to their diet (24.9% regular and 32.2% occasional use). In multivariable logistic regression, female gender, younger age, secondary educational level, place of residence (living in cites <100,000 residents of cities > = 500,000 residents), having at least 3 infections per year, lack of significant interest in own diet, and diet with restrictions on carbohydrate intake were significantly associated (*p* < 0.05) with higher odd of the regular use of dietary supplements in the last 3 months preceding the survey.

**Conclusion:**

This study revealed that the majority of working-age adults in Poland use dietary supplements, but only one-third of dietary supplement users consult with a doctor. Gender, age, educational level, place of residence, health status, and diet-related behaviors were significantly associated with attitudes towards the use of dietary supplements. There is a need for educational activities in the field of nutritional education and building public awareness about the indications for the use of dietary supplements.

## Introduction

Dietary supplements are products that are concentrated sources of vitamins, minerals, or other substances with a nutritional or physiological effect (1, 2). Dietary supplements are consumed to supplement the regular diet (1). Dietary supplements may support adequate nutrient intake in specific populations (e.g., in pregnant women or people on a diet that excludes the consumption of selected food groups) (2–4). Doctors may recommend the consumption of dietary supplements to prevent nutritional deficiencies and the development of some health conditions (2, 5). However, inappropriate or excessive use of dietary supplements may pose a health risk and lead to nutrient imbalances (6, 7). Unsupervised use of dietary supplements may also complicate public health messaging on balanced diets and nutritional principles (2, 5, 8).

The global prevalence of the use of dietary supplements has increased over the last two decades (9). Growing consumer awareness of nutrition, preventive health behaviors, and marketing influences are considered the major factors that shaped social behaviors related to the growing popularity of dietary supplements (1, 10).

According to the law that is in force in the European Union (EU), dietary supplements are regulated under the harmonized legislative framework (11, 12). Composition, labeling, and marketing of dietary supplements are regulated at the EU level, but authorization procedures and enforcement are regulated at the country level (11, 12).

In the EU, the prevalence of dietary supplement use varies substantially across countries (11, 13). It is estimated that in 2020, half of adults in Europe declared the use of at least one dietary supplement in the last 12 months (13). Vitamin D, vitamin C, magnesium, multivitamins and mineral combinations, and omega-3 are considered the most common dietary supplements used in Europe (13). High prevalence of dietary supplements is observed in Eastern Europe, especially the Czech Republic, Poland, Slovenia, and Romania (13). Demographic, socioeconomic, and cultural factors were described in the scientific literature as those that may shape individual behaviors towards the use of dietary supplements (14, 15). In society, the use of dietary supplements is often perceived as a part of a healthy lifestyle, especially in people who care about their health, practice sports, are better educated, and are more wealthy (2, 5).

Poland is one of the rapidly growing dietary supplements markets (11, 16). Introducing a new dietary supplement to the market only requires notification to the Chief Sanitary Inspectorate and is not subject to quality control or composition verification (16). The ease of introducing dietary supplements to the market in Poland means that there are over 30,000 different dietary supplements available for sale (11). Dietary supplements are advertised in mass media as well as in the digital media, with pharmacies, retail stores, and e-commerce platforms as the main sales channels (17, 18). The widespread marketing of dietary supplements leads to unauthorized consumption of dietary supplements in different sociodemographic groups in Poland.

Despite the growing popularity of dietary supplements, there is a limited number of population-based studies on the use of dietary supplements in Poland (19–22). Most of the studies are focused on particular subgroups, including students, healthcare professionals, older adults, and those with chronic diseases (11, 19). Stoś et al. (20) assessed the use of particular types of dietary supplements in a representative sample of Poles (data were collected from July 2019 to February 2020). Strocka et al. (19) assessed the awareness of dietary supplements in a representative sample of adults in Poland, with a focus on knowledge sources and purchasing habits.

There is a lack of up-to-date, representative epidemiological studies on the use of dietary supplements in Poland, after the COVID-19 pandemic. There is also a gap in research on the use of dietary supplement after the consultation with doctors. Adults of working age are of particular interest to public health, due to their contribution to the national economy related to occupational activities. A comprehensive understanding of nutrition-related inequalities and health behaviors in the Polish population is necessary to plan and develop health policies and educational strategies on nutrition and diet. Therefore, this study aimed to identify sociodemographic differences in the use of dietary supplements in a representative sample of Poles aged 18–64 years.

## Materials and methods

### Study design and data source

This is a secondary analysis of nationwide data from a cross-sectional survey on health-related behaviors of adults of working age in Poland. Data were acquired from the National Centre for Health Policy and Health Inequalities of the Cardinal Stefan Wyszynski University, which performed a nationwide cross-sectional survey called “Health prevention and health inequalities” (23) and shared the dataset for scientific use. As the original dataset was generated under the contract with the Polish Ministry of Education and Science (Agreement No. MEiN/2023/DPI/2717 of 13/10/2023), the Center offered free-of-charge data sharing for scientific purposes.

An official request was submitted to the National Centre for Health Policy and Health Inequalities, and a database covering a raw dataset of 12 questions was obtained.

### Population

As published by the National Centre for Health Policy and Health Inequalities, the original data came from a cross-sectional survey carried out between 4 and 16 December 2024, on a representative sample of 5,006 adults in Poland (23, 24). The study was carried out among adults of working age (18–64 years) (23). Quota sampling was applied. The study sample size was calculated following the stratification model that included: gender, age, size of the place of residence, and level of education. Demographic reports published by the Statistics of Poland were used to calculate the study sample. Data were weighted to reflect the structure of the working-age adult population (18–64 years) in Poland.

A dedicated public opinion pool company [ARC Rynek i Opinia (24)] was contracted to collect representative data on behalf of the National Centre for Health Policy and Health Inequalities. Respondents were recruited from the dataset managed by the public opinion pool company. The computer-assisted web interview technique (CAWI) was applied, and the study questionnaire was available online via a dedicated IT system. Respondents were obligated to answer all questions, so there were no missing answers. If the respondent refused to participate in the study, the next respondent who met the demographic criteria was invited to participate in the study (replacement of non-respondents). Each participant of the cross-sectional survey declared informed consent and voluntary willingness to participate in the study.

The study protocol of this retrospective analysis was reviewed and approved by the Ethical Committee at the Medical University of Warsaw (decision number: AKBE/56/2025).

### Measures

History of the use of dietary supplements for at least 3 months was defined based on the following question: “Have you taken any vitamins, supplements, minerals, or herbal products in the last 3 months?,” with three possible answers: no; yes, regularly; yes, occasionally.

The following question was addressed to respondents, who declared use of dietary supplements regularly or occasionally: “Were the supplements you are taking prescribed by a doctor?,” with three possible answers: yes, all of them; yes, but only some; no.

Nine questions on socio-demographic characteristics were used, including gender, age, educational background, place of residence, occupational status, marital status, self-declared health status, presence of chronic diseases, and history of infections in the last 12 months.

Moreover, questions on dietary habits were addressed (Supplementary File S1).

### Data analysis

Data obtained from the Centre for Health Policy and Health Inequalities were used to construct datasets for statistical analysis (23, 25). Analyses were performed using SPSS statistical software version 29 (IBM, Armonk, NY, US). Descriptive statistics were presented as frequencies and proportions. Differences between categorical variables were assessed using the chi-squared test.

A multivariable logistic regression model was developed to identify factors associated with the regular use of dietary supplements in the last 3 months (dependent variable). The selection of variables for the model based on theoretical assumptions. Variables describing the respondent’s socio-demographic status were included, as well as additional factors that might justify supplementation, such as a meatless diet, frequent infections, etc. All variables were incorporated into the model using a series of 0–1 variables (dummy variables). Model performance was evaluated using the Cox and Snell *R*^2^ and Nagelkerke *R*^2^ statistics. The Hosmer and Lemeshow test was conducted to assess goodness-of-fit. The result was *p* = 0.393, meaning the model fit was good. Associations were expressed as odds ratios (OR) with corresponding 95% confidence intervals (95% CI). Statistical significance was defined as *p* < 0.05.

## Results

In the analyzed dataset (n = 5,006), the share of women and men was similar: 49.9 and 50.1%, respectively. The mean age was 41.8, with a median of 42. Among the respondents, 47.1% reported no chronic diseases. Characteristics of the study population by the use of dietary supplements in the 3 months preceding the survey is presented in Table 1.

**Table 1:** Characteristics of the study population by the use of dietary supplements (*n* = 5,006).

	Use of dietary supplements in the last 3 months			
	Overall	No	Yes, occasionally	Yes, regularly
	*n* (%)	*n* (%)	*n* (%)	*n* (%)
Total	5,006 (100)	1,473 (100)	1,578 (100)	1955 (100)
Gender				
Male	2,506 (50.1)	852 (57.8)	792 (50.2)	862 (44.1)
Female	2,500 (49.9)	621 (42.2)	786 (49.8)	1,093 (55.9)
Age group				
18–24	541 (10.8)	135 (9.2)	192 (12.2)	214 (10.9)
25–34	989 (19.8)	266 (18.1)	322 (20.4)	401 (20.5)
35–44	1,326 (26.5)	384 (26.1)	397 (25.2)	545 (27.9)
45–64	2,150 (42.9)	688 (46.7)	667 (42.3)	795 (40.7)
Educational level				
Primary or vocational	1,593 (31.8)	599 (40.7)	483 (30.6)	511 (26.1)
Secondary	1895 (37.9)	523 (35.5)	598 (37.9)	774 (39.6)
Higher	1,518 (30.3)	351 (23.8)	497 (31.5)	670 (34.3)
Place of residence				
Rural area	2035 (40.6)	710 (48.2)	620 (39.3)	705 (36)
City <100,000	1,582 (31.6)	418 (28.4)	510 (32.3)	654 (33.4)
City 100,000-499,000	789 (15.8)	197 (13.4)	262 (16.6)	330 (16.9)
City > = 500,000	602 (12.0)	148 (10.0)	187 (11.8)	267 (13.7)
Employment				
Yes, full-time	2,863 (57.2)	842 (57.1)	878 (55.6)	1,143 (58.5)
Yes, part-time	902 (18.0)	197 (13.4)	300 (19.0)	405 (20.7)
No	1,242 (24.8)	435 (29.5)	400 (25.3)	407 (20.8)
Self-assessment of financial situation				
We have enough for everything and we are saving for the future	999 (20.0)	285 (19.4)	278 (17.6)	436 (22.3)
We have enough for everything without any special sacrifices, but we are not saving for the future	977 (19.5)	305 (20.7)	307 (19.5)	365 (18.7)
We live frugally and therefore have enough for everything	1804 (36)	494 (33.6)	600 (38.0)	710 (36.3)
We live very frugally to save for major purchases	678 (13.5)	182 (12.4)	228 (14.4)	268 (13.7)
We only have enough money for basic needs or we do not have enough money for even the cheapest food	547 (10.9)	206 (14.0)	165 (10.5)	176 (9.0)
Self-reported health status compared to peers				
Definitely better or slightly better	1,342 (26.8)	366 (24.8)	377 (23.9)	599 (30.6)
The same or hard to say	2,323 (46.4)	740 (50.2)	751 (47.6)	832 (42.6)
A little worse	1,003 (20.0)	263 (17.9)	343 (21.7)	397 (20.3)
Definitely worse	338 (6.8)	104 (7.1)	107 (6.8)	127 (6.5)
How often do you get sick/cold?				
Not at all (never sick)	460 (9.2)	208 (14.1)	94 (6.0)	158 (8.1)
1–2 times a year	3,125 (62.4)	942 (64.0)	978 (62.0)	1,205 (61.6)
3–4 times a year	1,057 (21.1)	244 (16.6)	362 (23.0)	451 (23.1)
5 times a year or more	363 (7.3)	79 (5.4)	143 (9.1)	141 (7.2)
Number of chronic diseases diagnosed by a doctor				
No disease	2,359 (47.1)	797 (54.1)	723 (45.8)	839 (42.9)
1 disease	1,282 (25.6)	354 (24)	443 (28.1)	485 (24.8)
2 diseases	699 (14.0)	187 (12.7)	213 (13.5)	299 (15.3)
3 or more	666 (13.3)	135 (9.2)	199 (12.6)	332 (17)

**Figure 1:**
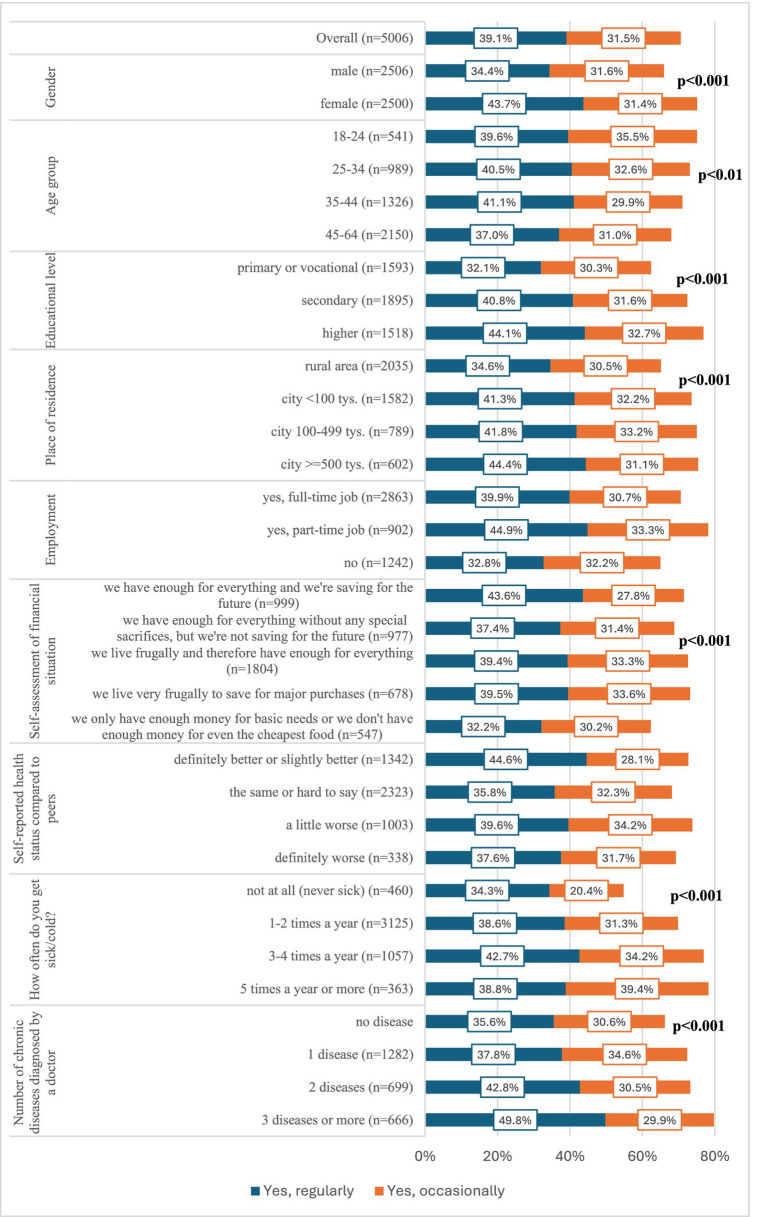
Percentage of respondents who declared taking dietary supplements in the last 3 months, depending on socio-demographic factors and health status (*n* = 5,006).

Among all respondents (*n* = 5,006), 39.1% reported regular use of dietary supplements in the 3 months preceding the present study, and another 31.5% reported occasional use (Figure 1). In men, these percentages were 34.4 and 31.6%, respectively, and in women, 43.7 and 31.4% (*p* < 0.001) (Figure 1). There were also statistically significant differences in the frequency of dietary supplements use by age (*p* < 0.01), educational level (*p* < 0.001), place of residence (*p* < 0.001), financial situation (*p* < 0.001), number of infections per year (p < 0.001) and number of chronic diseases diagnosed (*p* < 0.001) (Figure 1). Details are presented in Figure 1.

Among those who regularly follow a box diet (*n* = 97), 56.7% declared the regular use of dietary supplements in the 3 months preceding the survey, and 21.6% declared occasional use of dietary supplements, compared (*p* < 0.01) to 38.7% regular and 31.7% occasional users of dietary supplements among those among those who did not follow a box diet (Table 2). Intermittent fasting (*n* = 297) reported using supplements regularly in 44.4% of cases, and occasionally in 34.3% (*p* < 0.01). Among those who avoided meat (*n* = 455), 51.9% used supplements regularly, and 31.6% occasionally (*p* < 0.001). Among those who reported following a low-carbohydrate diet (*n* = 517), 58.4% reported regular supplement use, and 27.9% reported occasional supplement use (*p* < 0.001), for a total of 86.3%. This was the highest score obtained for dietary behaviors. Among those who reported limiting their animal fat intake (*n* = 641), 53.8% also reported regular supplement use, and 28.7% reported occasional supplement use (*p* < 0.001). Supplement use in the last 3 months was least common among those who reported not paying much attention to their diet (*n* = 1808). Of this group, 24.9% reported regular supplement use, and 32.2% reported occasional supplement use (*p* < 0.001), for a total of 57.1%. Detailed data are presented in Table 2.

**Table 2:** Eating behaviors* and supplement use in the last 3 months.

Self-declared eating behaviors	Use of dietary supplements in the last 3 months			
	No	Yes, occasionally	Yes, regularly	*p*
I follow a box diet (*n* = 97)	21.6%	21.6%	56.7%	**<0.01**
I practice intermittent fasting (*n* = 297)	21.2%	34.3%	44.4%	**<0.01**
I reduce sugar in my food and drinks (*n* = 1,447)	16.9%	30.3%	52.7%	**<0.001**
I reduce salt in my food (*n* = 878)	15.9%	31.9%	52.2%	**<0.001**
I avoid eating meat (*n* = 455)	16.5%	31.6%	51.9%	**<0.001**
I limit carbohydrates (low-carb diet) (*n* = 517)	13.7%	27.9%	58.4%	**<0.001**
I reduce animal fat in my food and drinks (*n* = 641)	17.5%	28.7%	53.8%	**<0.001**
I make sure to include whole grains in my diet (*n* = 982)	17.0%	28.8%	54.2%	**<0.001**
I strive for a high protein diet (*n* = 897)	16.8%	30.2%	53.0%	**<0.001**
I deliberately choose vegetable fats (e.g., olive oil, rapeseed oil, flaxseed oil) (*n* = 679)	16.1%	26.1%	57.9%	**<0.001**
I make sure to include vegetables in my daily diet (*n* = 1,683)	18.5%	31.2%	50.3%	**<0.001**
I make sure to include dietary fiber in my daily diet (*n* = 876)	16.7%	28.0%	55.4%	**<0.001**
I eliminate products containing preservatives/artificial colors (*n* = 1,032)	15.5%	30.1%	54.4%	**<0.001**
I do not pay much attention to my diet (*n* = 1808)	42.9%	32.2%	24.9%	**<0.001**
Total (*n* = 5,006)	29.4%	31.5%	39.1%	–

*The table presents only data for people declaring specific dietary behaviors. Statistically significant values are bolded.

Among those who used supplements in the 3 months preceding the study (Table 3), 11.4% had all of their supplements prescribed by a doctor, and another 22.7% had some of them. The remaining 65.9% used supplements without consulting a doctor. Women were slightly more likely than men to consult a doctor about taking supplements (*p* < 0.05). Among women who took supplements, 11.8% had all of their supplements prescribed by a doctor, and another 24.3% had some of them. Among men, the figures were 11.1 and 20.8%, respectively. There were no statistically significant differences by age (*p* = 0.088), education (*p* = 0.673), size of town of residence (*p* = 0.994), and financial situation (*p* = 0.086). Among those who rated their health as significantly worse than their peers (*n* = 234) and used supplements, 14.5% used all prescribed supplements, and a further 29.5% used some of them. By comparison, among those who rated their health as significantly or slightly better than their peers (*n* = 976), the figures were 12.2 and 23.3%, respectively (*p* < 0.01). Among those who reported being sick five or more times a year (*n* = 284), 14.1% used all prescribed supplements, and 27.5% used some of them compared to 12.7 and 19% among those who were not sick at all (*p* < 0.01). Among those diagnosed with three or more chronic diseases and taking supplements (*n* = 532), 15.2% had all of them prescribed by a doctor, and 34.4% used some of them. Among those without any diagnosed chronic disease (*n* = 1,563), the figures were 9.5 and 18.0%, respectively (*p* < 0.001). Detailed data are presented in Table 3.

**Table 3:** Consulting a doctor about the use of supplements - respondents’ declarations regarding the supplements used in the last 3 months preceding the survey, depending on socio-demographic factors and health status.

Were the dietary supplements you are taking prescribed by a doctor?					
	*n*	Yes, all of them	Yes, but only some	No	*p*
Total	3,534	11.4%	22.7%	65.9%	–
Gender					
Male	1,655	11.1%	20.8%	68.2%	**<0.05**
Female	1879	11.8%	24.3%	63.9%	
Age group					
18–24	406	14.5%	21.7%	63.8%	0.088
25–34	723	12.4%	22.4%	65.1%	
35–44	942	11.7%	20.9%	67.4%	
45–64	1,462	9.8%	24.2%	65.9%	
Educational level					
Primary or vocational	994	11.5%	22.4%	66.1%	0.673
Secondary	1,373	11.8%	21.6%	66.6%	
Higher	1,167	11.0%	24.1%	65.0%	
Place of residence					
Rural area	1,325	11.0%	23.1%	65.9%	0.994
City <100,000	1,164	11.6%	22.4%	66.0%	
City 100,000-499,000	592	11.5%	22.8%	65.7%	
City > = 500,000	453	12.1%	21.9%	66.0%	
Employment					
Yes, full-time	2020	11.1%	22.2%	66.7%	**<0.01**
Yes, part-time	706	11.8%	27.2%	61.0%	
No	807	12.0%	19.7%	68.3%	
Self-assessment of financial situation					
We have enough for everything and we are saving for the future	713	13.6%	21.7%	64.7%	0.086
We have enough for everything without any special sacrifices, but we are not saving for the future	672	10.6%	26.2%	63.2%	
We live frugally and therefore have enough for everything	1,310	10.1%	22.2%	67.7%	
We live very frugally to save for major purchases	496	12.3%	22.8%	64.9%	
We only have enough money for basic needs or we do not have enough money for even the cheapest food	342	12.6%	19.3%	68.1%	
Self-reported health status compared to peers					
Definitely better or slightly better	976	12.2%	23.3%	64.5%	**<0.01**
The same or hard to say	1,583	10.0%	21.0%	69.0%	
A little worse	740	12.4%	23.2%	64.3%	
Definitely worse	234	14.5%	29.5%	56.0%	
How often do you get sick/cold?					
Not at all (never sick)	252	12.7%	19.0%	68.3%	**<0.01**
1–2 times a year	2,183	10.4%	21.6%	68.0%	
3–4 times a year	813	12.8%	25.1%	62.1%	
5 times a year or more	284	14.1%	27.5%	58.5%	
Number of chronic diseases diagnosed by a doctor					
No disease	1,563	9.5%	18.0%	72.5%	**<0.001**
1 disease	928	11.7%	20.9%	67.3%	
2 diseases	511	12.9%	27.8%	59.3%	
3 or more	532	15.2%	34.4%	50.4%	

Statistically significant values are bolded.

A multivariable logistic regression model (Table 4) predicting regular use of dietary supplements in the last 3 months preceding the survey yielded a Cox-Snell R-square of 0.070 and a Nagelkerke *R*-square of 0.095. Men had lower odds (aOR = 0.79; 95%CI: 0.70–0.89; *p* < 0.001) of regular use of dietary supplements in the 3 months preceding the survey.

**Table 4:** Multivariable logistic regression model predicting regular use of dietary supplements in the last 3 months preceding the survey (n = 5,006).

	aOR (95%CI)	*p*
Gender		
Male	0.79 (0.70–0.89)	**<0.001**
Female	Reference	Reference
Age group		
18–24	1.28 (1.04–1.57)	**<0.05**
25–34	1.18 (0.99–1.39)	0.06
35–44	1.21 (1.04–1.41)	**<0.05**
45–64	Reference	Reference
Educational level		
Primary or vocational	Reference	Reference
Secondary	1.19 (1.03–1.38)	**<0.05**
Higher	1.17 (0.99–1.39)	0.057
Place of residence		
Rural area	Reference	Reference
City <100,000	1.22 (1.06–1.41)	**<0.01**
City 100,000-499,000	1.17 (0.98–1.40)	0.076
City > = 500,000	1.23 (1.01–1.50)	**<0.05**
How often do you get sick/cold?		
Not at all (never sick)	Reference	Reference
1–2 times a year	1.03 (0.89–1.2)	0.669
3–4 times a year	1.23 (1.03–1.47)	**<0.05**
5 times a year or more	1.55 (1.28–1.87)	**<0.001**
Number of chronic diseases diagnosed by a doctor		
No disease	Reference	Reference
1 disease	1.08 (0.87–1.34)	0.483
2 diseases	1.14 (0.90–1.45)	0.28
3 or more	0.99 (0.73–1.34)	0.941
I do not pay much attention to my diet		
Yes	0.46 (0.40–0.53)	**<0.001**
No	Reference	Reference
I follow a so-called box diet		
Yes	1.51 (1.00–2.29)	0.052
No	Reference	Reference
I practice intermittent fasting		
Yes	0.81 (0.63–1.04)	0.095
No	Reference	Reference
I avoid eating meat		
Yes	1.18 (0.96–1.45)	0.111
No	Reference	Reference
I limit carbohydrates (low-carb diet)		
Yes	1.71 (1.40–2.07)	**<0.001**
No	Reference	Reference

Statistically significant values are bolded.

Age 18–24 was associated with higher odds of regular use of dietary supplements when compared to individuals aged 45–64 (aOR = 1.28; 95%CI: 1.04–1.57; *p* < 0.05). Living in the largest cities over 500,000 inhabitants (aOR = 1.23; 95%CI:1.01–1.50; *p* < 0.05) or cities <100,000 residents (aOR = 1.22; 95%CI:1.06–1.41; *p* < 0.01) was also associated with higher odds of regular use of dietary supplements. Experiencing infections at least three times a year was significantly associated with higher odds of regular use of dietary supplements (*p* < 0.05).

Those who reported a lack of significant interest in their diet had lower odds of regular use of dietary supplements (aOR = 0.46; 95%CI: 0.40–0.53; *p* < 0.001). Those who restricted their carbohydrate intake had higher odds of regular use of dietary supplements (aOR = 1.71; 95%CI: 1.40–2.07; *p* < 0.001). Detailed data are presented in Table 4.

## Discussion

This is the first nationwide representative cross-sectional survey on dietary supplement use among adults of working age in Poland. This study revealed high use of dietary supplements in Poland. However, only one-third of dietary supplement users consulted a doctor about supplement use, and the list of supplements taken was provided by a doctor. The highest percentage of adult who used dietary supplements was observed in the carbohydrate-restricted group (86.3%), and the lowest (57.1%) among those who did not pay attention to their diet. In multivariable logistic regression, gender, age, educational level, place of residence, number of infections per year, lack of significant interest in own diet, and diet with restrictions on carbohydrate intake were significantly associated with the regular use of dietary supplements in the last 3 months preceding the survey.

Findings from this study revealed high consumption of dietary supplements among adults of working age in Poland, with 39.1% of regular users and 31.5% of occasional users. This observation confirms high consumption of dietary supplements in Poland (13). The prevalence of dietary supplement use in Europe (at least occasionally) is estimated at 40–60% of the population (10, 11, 13). National regulations on dietary supplements, including market access and marketing, may have a significant impact on the prevalence of dietary supplement use in different EU countries (26). As there is a lack of other representative data on the prevalence of dietary supplements in Poland, after the COVID-19 pandemic, direct comparisons with other studies are not possible.

Findings from this study provided data on socio-demographic differences in the prevalence of dietary supplement use in Poland. Females were more likely to use dietary supplements, which is in line with previously published data in the scientific literature (27, 28). It is believed that females are more often present with pro-healthy behaviors and higher healthcare utilization (29). Moreover, females might consume more dietary supplements related to beauty and skin care (30). In this study, the prevalence of use of dietary supplements decreased with age. This observation is contrary to the previously published data, when older adults were more likely to use dietary supplements (31, 32). This observation may result from the fact that younger adults might be more exposed to dietary supplements marketing on social media and e-commerce shops (33).

Secondary education was associated with higher odds of regular use of dietary supplements. Higher education might be associated with greater critical appraisal of supplement claims. Moreover, different educational groups are targeted by different marketing channels. When compared to residents of rural areas, those who lived in the smallest cities (below 100,000 residents), but also inhabitants of the largest cities (over 500,000 residents), were more likely to regularly use dietary supplements. This observation suggests that place of residence plays an important role in shaping individuals’ behaviors related to health (34). Residents of urban areas may be exposed to a higher number of marketing campaigns for dietary supplements, including posters and billboards. Moreover, living in urban areas may lead to a higher interest in health and westernization of lifestyle, with dietary supplementation perceived as a part of pro-healthy choices (35). Moreover, those who live in urban areas may have higher access to dietary supplements in retails stores and pharmacies. Moreover, they can be also exposed to marketing in public places—including banners and leaflets at the bus stations. Younger age (18–24) was associated with higher odds of supplement use. This observation may result from the widespread promotion of dietary supplement in social media, especially Instagram and YouTube in Poland. However, this finding requires further investigation.

There was a lack of significant impact of self-reported economic status on dietary supplement intake, which may result from the fact that dietary supplements are available in different formulations and prices, which are accessible to each economic group (9, 11).

Frequent infections (at least 3 a year) are linked to higher supplement use is crucial. This observation suggest, that individuals in Poland are likely self-medicating with supplements based on perceived immune benefits, often without medical guidance. There is an urgent need to educate general public about the dietary supplements. Common myths and non-evidence based theories on dietary supplements use during infections should be explained by health professionals. In this study, those who got sick at least 3 times a year declared higher consumption of dietary supplements. This may result from the fact that those who often get sick consume dietary supplements to boost their immunity. However, the impact of dietary supplements on the immune system is often based on low-quality scientific evidence, and marketing strategies of dietary supplements may lead to a misleading perception among society (17, 33).

Dietary supplements should be used in order to provide substances (including vitamins and microelements) that are not present in the diet in a sufficient quantity (1, 2). People who follow a particular diet, especially exclusion diets, e.g., vegetarians or vegans, constitute groups that, due to eating behaviors, should supplement some dietary elements. In this study, those who followed particular diets more often used dietary supplements. The lowest percentage of dietary supplement users was among those who did not pay much attention to their diet. This observation is in line with expectations that those who take care of their diet are more likely to use dietary supplements, as these groups might be more aware of nutritional guidelines and the role of particular vitamins and microelements in the diet. Low-carbohydrate diet was associated with higher consumptions of dietary supplements. We can hypothesize that this group of Poles pay more attention to their food behaviors and check the nutritional values of their meals, so maybe more aware of deficiencies in their diet. However, this observations requires further investigation.

Strocka et al. (19) reported high awareness of dietary supplements among Polish adults in 2024, with significant differences based on gender and education, as well as the Internet as the major source of knowledge on dietary supplements. Kołodziej et al. (36) reported that a significant percentage of Poles (October 2017 and July 2018) demonstrate inaccurate information about dietary supplements. In this study, only 34.1% of dietary supplement users consulted a doctor about supplementation. Among those who used supplements in the 3 months preceding the study, 11.4% had all of their supplements prescribed by a doctor, and another 22.7% had some of them. Similar results were observed in other countries, with a significant percentage of the population who used dietary supplements on their own, without consultation with healthcare professionals (37). This phenomenon may lead to inappropriate use of dietary supplements, interactions with medications, and pose a health risk (38).

Use of dietary supplements without consultation with a doctors may lead to, e.g., interactions with medications, excessive intake, wasted resources. There are different information sources on dietary supplements like pharmacists or the Internet, that people may be using instead of physicians. Dietary supplementation under the doctor’s supervision is crucial to enhance the health effects of supplementation and reduce risk of adverse effects and interactions with medicines.

### Practical implications

This study has practical implications for public health in Poland. This study revealed high consumption of dietary supplements in Poland, with a low percentage of dietary supplement users who consult with doctors about the supplements they take. This observation underlined the need for educational campaigns and building awareness on dietary supplements among adults in Poland. This study also revealed sociodemographic differences in the use of dietary supplements, with gender, age, educational level, place of residence, number of infections per year, and eating behaviors as factors associated with the use of dietary supplements. These observations underline that behaviors related to dietary supplements vary between different socio-demographic groups, and personalized communication on dietary supplement use is needed. Primary care physicians should pay more attention to talking with patients about the dietary supplements and medical conditions that will justify the use of dietary supplements. This study also underlines the need for nutritional education, targeted to different sociodemographic groups, especially among adults of working age.

Further studies should include longitudinal follow-up observations. Moreover, study based on medical records analyses are needed for greater accuracy.

### Limitations

This study represents a secondary analysis of data derived from a cross-sectional survey conducted using computer-assisted web interviewing (CAWI), which constitutes the primary limitation of the research. In Poland, 96% of households has an Internet access (39). Data on the use of dietary supplements were self-declared. The use of dietary supplements was measured using a single 3-month recall, which is a limitation of this study. However, self-reported use is a standard question in cross-sectional surveys, including those on dietary supplements. Due to the high number of dietary supplements available on the market, questions on particular brands of supplements are not possible. Further studies should provide a more precise assessment of dietary supplement intake. There were no questions on the use of particular dietary supplements (e.g., vitamins B, D, C, microelements, magnesium, etc.), which is a limitation of this study. Further studies should collect these information. Medical records were not verified to check whether the doctors recommended dietary supplement intake. There are different approaches to multivariable analysis that might be implemented. Cross-sectional design precludes causal inference. Sensitivity analyses were not performed. Factors like marketing influence, social media use, specific health beliefs were not included in this analysis.

## Conclusion

This study revealed that the majority of working-age adults in Poland use dietary supplements, but only one-third of dietary supplement users consult with a doctor. Females, younger adults, those with secondary education, inhabitants of cities <100,000 residents or the largest cities with more than 500,000 residents, respondents who had at least 3 infections (got sick) per year, as well as those who limit carbohydrates (low-carb diet) in their diet, are more likely to use dietary supplements regularly. Due to the high percentage of the population using dietary supplements without consulting a doctor, there is a need for educational activities in the field of nutritional education and building public awareness about the indications for the use of dietary supplements. Sociodemographic groups with high consumption of dietary supplements without consultation with doctors should be targeted as priority population for educational campaigns.

## Data Availability

The datasets presented in this article are not readily available because the authors do not have the rights to share the data publicly. The datasets are available from the corresponding author, upon reasoned request. Requests to access these datasets should be directed to r.sierpinski@uksw.edu.pl.
