# Supplementary material for: Sociodemographic differences in the use of dietary supplements in a representative sample of adults in Poland—a secondary analysis
Source: Front Nutr. 2025 Dec 16;12:1724264. doi: 10.3389/fnut.2025.1724264 (PMC12751291; doi:10.3389/fnut.2025.1724264)
Supplement: Supplementary file 1 [file Table_1.DOCX]

P1. Respondent's gender:

a) Male

b) Female

P2. In what year were you born? _ _ _ _

P3. What is your education level? Please provide the highest level of education you have achieved.

a) rural area

b) city below 100,000 residents

c) city 100,000-499,000 residents

d) city >=500,000 residents

P4. What is your education level? Please provide the highest level of education you have achieved.

e) Unfinished primary or no school education

f) Primary

g) Lower secondary

h) Basic vocational (also SPR)

i) General secondary without matriculation exam

j) General secondary with matriculation exam

k) Vocational secondary without matriculation exam

l) Vocational secondary with matriculation exam (technical school, vocational or technical high school)

m) Post-secondary or post-secondary

n) Higher education with the title of engineer, bachelor, certified economist

o) Higher education with a master's degree, medical degree or equivalent

p) Higher education with a doctoral degree or higher

P5. Are you currently gainfully employed (full-time, in your own company or farm or do you perform commissioned work)?

a) Yes, full-time

b) Yes, part-time

c) Yes, occasional

d) No

P6. Which of the following best describes the financial situation of your household?

a) We have enough for everything and we are also saving for the future

b) We have enough for everything without any special sacrifices but we are not saving for the future

c) We live frugally and thanks to this we have enough for everything

d) We live very frugally to save for more serious purchases

e) We only have enough money for basic needs

f) We do not have enough money even for the cheapest food

P7. Do you suffer from any of the following diseases? [YES/NO/I DON'T KNOW - for each]

a) Allergies (e.g. skin allergies, hay fever)

b) Food intolerance, food allergy

c) Urinary tract diseases (urolithiasis, kidney failure, glomerulonephritis, other kidney or urinary tract diseases)

d) Circulatory system diseases (hypertension, previous stroke, previous myocardial infarction, coronary artery disease, heart failure, atherosclerosis of the lower limb arteries, lipid disorders (high: total cholesterol, LDL, triglycerides), other heart diseases)

e) Musculoskeletal diseases (joint disease, osteoporosis, other)

f) Type 1 diabetes

g) Type 2 diabetes

h) Digestive system diseases (peptic ulcer disease, gastroesophageal reflux disease, bowel disease, liver disease, pancreas disease, gallstone disease biliary, other)

i) Respiratory diseases (COPD, tuberculosis, asthma, other)

j) Endocrine diseases (hypothyroidism, hyperthyroidism, other)

k) Neurological diseases (Parkinson's disease, multiple sclerosis, other)

l) Cancer

m) Skin diseases (including psoriasis)

n) None of the above

P8. On average, how often during the year are you sick or have a cold, excluding situations resulting from a chronic disease)

a) I never get sick

b) 1-2 times a year

c) 3-4 times a year

d) 5-6 times a year

e) 7-12 times a year

f) More than 12 times a year

P9. Is my health in comparison to people my age?

a) Definitely better

b) A little better

c) The same

d) A little worse

e) Definitely worse

f) Hard to say

P10. Which of the following best describes your daily eating habits? [MULTIPLE CHOICE]

a) I don't pay much attention to my diet

b) I follow the so-called Box diet

c) I practice intermittent fasting

d) I reduce the sugar content in my food and drinks

e) I reduce the salt content in my food

f) I avoid eating meat

g) I limit carbohydrates (low-carb diet)

h) I reduce the animal fat content in my food and drinks

i) I ensure the presence of whole grain products in my diet

j) I strive for a high protein content in my diet

k) I deliberately choose vegetable fats (e.g., olive oil, rapeseed oil, flaxseed oil)

l) I ensure the presence of vegetables in my daily diet

m) I ensure the presence of dietary fiber in my daily diet

n) I eliminate products containing preservatives/artificial colors

o) None of the above

P11. Have you taken any vitamins, supplements, minerals, or herbal products in the last 3 months?

a) No

b) Yes, regularly

c) Yes, occasionally

FILTER: YES

P12. Were the supplements you are taking prescribed by a doctor?

a) Yes, all of the supplements I take

b) Yes, but only some

c) No
